# The assessment of fukuda stepping test results in prognosis of benign paroxysmal postural vertigo

**DOI:** 10.1016/j.bjorl.2021.05.005

**Published:** 2021-06-01

**Authors:** Işıl Taylan Cebi, Abdullah Karatas

**Affiliations:** Haseki Training and Research Hospital, Clinic of Otorhinolaryngology, Istanbul, Turkey

**Keywords:** Intractable vertigo, Fukuda stepping test, Unterberger, Unilateral vestibular dysfunction

## Abstract

**Introduction:**

Benign paroxysmal postural vertigo originating from the peripheral vestibular system is characterized by brief vertigo spells triggered by the sudden head motion. Usually, vestibular dysfunction in benign paroxysmal postural vertigo is unilateral. Fukuda stepping test which is helpful in the diagnosis of unilateral vestibular dysfunction, may also be valuable in the prediction of prognosis of benign paroxysmal postural vertigo.

**Objective:**

The purpose of this study is to evaluate the relevance of Fukuda stepping test results with resistant and/or recurrent benign paroxysmal postural vertigo cases.

**Methods:**

We evaluated 62 patients with unilateral, idiopathic benign paroxysmal postural vertigo of posterior and/or lateral canals. The Fukuda stepping test was performed prior to the Dix-Hallpike and head-roll tests. Two groups were created according to the Fukuda stepping test results. In Group 1 Fukuda stepping test results were positive with a deviation angle >45°, while in Group 2 the results were negative with no apparent deviation. Two groups were compared by the number of canalith repositioning manuevers performed and the frequency of recurrences.

**Results:**

We found Fukuda stepping test to be invaluable in the diagnosis of benign paroxysmal postural vertigo since the ratio of Fukuda stepping test positivity and negativity were similar in benign paroxysmal postural vertigo patients. However, the need for multiple canalith repositioning manuevers was significantly higher in Group 1 (*p* = 0.0103). In addition, the recurrence frequency was found significantly lower in the Group 2 (*p* = 0.0441).

**Conclusion:**

Although the sensitivity of Fukuda stepping test in detecting mild/moderate unilateral vestibular dysfunction is poor, it may be valuable in prediction of the prognosis of benign paroxysmal postural vertigo. We suggest that positive Fukuda stepping test results in benign paroxysmal postural vertigo patients indicate poor prognosis, the need for multipl canalith repositioning manuevers and the higher possibility of recurrences.

## Highlights

• Patients are relieved of benign paroxysmal postural vertigo symptoms after canalith repositioning manuevers however, some may be resistant.
• The need for multiple manuevers is more in Fukuda stepping test positive patients.
• The recurrence is less in Fukuda stepping test negative patients.
• When the Fukuda stepping test is positive, poor prognosis, need for multipl canalith repositioning manuevers and recurrences are expected.
• Fukuda stepping test is a valuable bedside test to predict the prognosis of benign paroxysmal postural vertigo.

## Introduction

Benign paroxysmal postural vertigo (BPPV) originating from the peripheral vestibular system is characterized by brief vertigo spells triggered by the sudden head motion.^1^ At present, the widely accepted theory about the pathophysiology is the separation of otoconia and debris from the neuroepithelium of utricular or saccular macula.^2^ The freely floating otoconia in the semicircular canals or those sticking to the cupula, provoke short-term nystagmus and vertigo.^3^ Because of the topography of semicircular canals, freely floating otoconia move especially into the posterior semicircular canal.^4^ The underlying pathology of the separation of otoconia from the neuroepithelium is obscure. According to some studies, separation happens due to the structural changes of the otoconia or the degenerative changes of the neuroepithelium of utricular or saccular macula and the ganglion cells of the saccular nerve. Therefore both saccular and utricular dysfunction may be observed in BPPV.^1^, ^5^

Usually, vestibular dysfunction in BPPV is unilateral. Various studies have detected the unilateral involvement in BPPV by vestibular evoked myogenic potential (VEMP) testing.^6^, ^7^, ^8^, ^9^

Vestibulo-ocular (VOR) and vestibulo-spinal (VSR) reflexes influenced by the vestibular end organs, help the stabilization of our vision, posture, and gait during the activities of daily life. While VOR assessment measures like caloric irrigation have been the gold standard for the identification of peripheral vestibular dysfunction, VSR measurements are also useful for the evaluation of labyrinthine dysfunction. In 1938 Unterberger proposed a stepping test with eyes shut for the diagnosis of unilateral vestibular dysfunction.^10^ The test was improved and popularized by Fukuda and named as Fukuda stepping test (FST) in 1959.^11^ In FST the patients are asked to stand upright, extend both arms and walk in place for 50–100 steps with eyes shut. Individuals with healthy vestibular function tend to walk forward with no rotation to either sides. In the presence of a vestibular dysfunction patients tend to rotate more than 45° to the side of the lesion.^11^

The majority of patients are quickly relieved of BPPV symptoms after canalith repositioning manuevers (CRM), and the prognosis is good. However, some cases may be resistant to standard treatment manuvers and the recurrences are seen commonly.

The purpose of this study is to evaluate the relevance of FST results with resistant and/or recurrent BPPV cases. We hypothesized that as severity of BPPV increases the overall diagnostic performance of the FST improves and FST may be a valuable bedside test to predict the prognosis of BPPV.

## Methods

This study was conducted between June and November 2019, prospectively. Sixty-two patients with unilateral, idiopathic BPPV of posterior or lateral canals were included. Of these patients selected, 35 were female and 27 were male, with an average age of 45.3 years (range 23–67 years). The diagnosis of BPPV and the side of the lesion were established by the patient history, the Dix-Hallpike test and the head-roll tests, according to the diagnostic guideline of the Committee for the Classification of Vestibular Disorders of the Barany Society.^12^ Patients with both sides involved or the lesion side could not be determined, patients with history of Meniere’s disease and/or other labyrinthopathies, recent trauma and inner ear diseases, patients who were unable to perform the FST and unable to tolerate the Dix-Hallpike and head-roll tests were excluded from the study group. Fifty-one posterior canal and 11 lateral canal BPPV cases were detected.

The FST was performed prior to the Dix-Hallpike and head-roll tests. The test was performed in a quiet room to prevent the patients from orienting to sound. Patients were asked to stand on a tiled floor, extend their arms parallel to the ground and walk in place for 50 steps, eyes shut. An examiner demonstrated the FST prior to testing so that the patients could fully understand the task. Also, the examiner stood close by in order to prevent the patients from falling during the test. Whether the patients opened their eyes, they were asked to stop and re-instructed to maintain eyes shut. Once the test was completed, the final degree and direction of deviation were recorded. The deviation angle was measured by a marked grid on the tile floor. A deviation angle >45° to the either side or a fall during the test were defined as abnormal.

After the FST, repositioning maneuvers (Epley, Barbecue) regarding the involved canal were performed. All patients were examined once a week and CRM were re-performed to those showing no improvement. Control examination and maneuvers were continued until the symptoms had disappeared. Repetition of BPPV after 3 months of symptom free interval was defined as recurrence.

Sixty-two BPPV patients were included in two groups according to FST results. In Group 1 FST results were positive with a deviation angle >45°, while in Group 2 the results were negative with no apparent deviation. The two groups were compared by the number of CRMs performed and the frequency of recurrences.

Statistical analyses were performed using statistical software (SPSS 22.0, SPSS Inc.; Chicago, IL, USA). A significant difference was defined as *p* < 0.05. The mean, standard deviation, median, minimum value, maximum value, frequency, and ratio were used for the definitive statistics of the data. Free samples *t*-test were used for the analysis of quantitative data. Fisher’s and Chi-Square tests were used for the analysis of qualitative data.

The design of our study was reviewed and approved by the local ethics committee (reference nº 06/2019). Oral and written informed consent about the design, aim, and clinical implications of the study was taken from all participants, according to the Declaration of Helsinki.

## Results

A total of 62 patients with unilateral BPPV were recruited to our study (35 females/27 males). The average age was 45.3 years (range 23–67 years). Of these patients 51 (83.6%) were posterior and 11 (16.4%) were lateral canal BPPV. Two groups were created due to FST results. Group 1 consisted of 33 (53.2%) patients having positive FST results and and Group 2 consisted of 29 (46.8%) patients with negative FST results. No statistically significant difference was found between the sex and age distribution of the Group 1 and Group 2, *p* = 0.7943 and *p* = 0.5262 respectively (Table 1). In addition, the ratio of FST positive and FST negative BPPV patients were not statistically significant.

**Table 1:** Definitional characteristics of Group1 and Group 2.

		Group 1		Group 2		
Age		Mean ± SD	Med (min–max)	Mean ± SD	Med (min–max)	*p*
		46.18 ± 11.11	45.5 (23–62)	44.41 ± 10.64	43 (27–67)	0.5262
Sex	M	20 (60.6%)		19 (65.5%)		0.7943
	F	13 (39.4%)		10 (34.5%)		
Posterior canal BPPV		26 (78.8%)		25 (86.2%)		0.5194
Lateral canal BPPV		7 (21.2%)		4 (13.8%)		
Total		33		29		

M, Male; F, Female.

Independent samples *t*-test, Fisher’s and Chi-Square test.

Of the thirtythree patients with positive FST results, 18 (54.5%) turned toward the lesion side and 15 (45.5%) turned toward the intact side. No significant relation between the direction of deviation and the side affected by BPPV was observed.

Group 1 and 2 were compared by the number of CRMs performed and the frequency of recurrences. In Group 1, 11 patients (33.3%) were treated by a single CRM and 22 patients (67.7%) needed multiple CRMs for recovery. In Group 2, 20 patients (68.9%) were treated by a single CRM and only nine patients (31.1%) needed multipl CRMs. The need for multiple CRMs was significantly higher in the FST positive group (*p* = 0.0103). Also, the recurrence frequency of the two groups were compared. In Group 1, 19 patients (57.6%) had recurrent BPPV after three months of recovery, 14 patients (42.4%) had no symptoms at all. In Group 2, 9 patients (31.1%) had a recurrence, and 20 patients (68.9%) were symptom free. When the two groups were compared recurrence, frequency was significantly lower in the FST negative group (*p* = 0.0441) (Table 2).

**Table 2:** Comparison of the two groups according to the number of CRMs performed and the frequency of recurrences.

	Group 1	Group 2	*p*
**CRM single**	11 (33.3%)	20 (68.9%)	0.0103
Multiple	22 (67.7%)	9 (31.1%)	
**Reccurences positive**	19 (57.6%)	9 (31.1%)	0.0441
Negative	14 (42.4%)	20 (68.9%)	

CRM, Canalith repositioning manuevers.

Fisher’s and Chi-Square test.

## Discussion

The three components of balance are proprioception, VOR and VSR. These together help the stabilization of our vision, posture, and gait during the activities of daily life. The otolith organs (utricle and saccule) are a component of VSR. Utricle and saccule maintain the perception of motion at horizontal and vertical planes, respectively.

The FST is based on vestibulospinal reflex and widely used for evaluation of labyrinthine function. Closing eyes during the FST deactivates the VOR and FST acts as a bedside test to assess the VSR and otolith organs. The previous study of Ben-David et al. has demonstrated that FST is more sensitive than the Romberg test in detection of unilateral vestibular asymmetry.^13^ FST duration and angle of rotation were found to be significantly increased in patients with peripheral vestibular pathology. Mc Caslin et al. reported the sensitivity of FST in unilateral vestibular hypofunction as 70%, which is similar to the findings (71%) of Moffat et al.^14^, ^15^ However the reliability of FST is controversial. In a recent study, the FST was not found to be reliable as a screening tool for peripheral vestibular asimmetry in chronically dizzy patients.^16^ In our study the ratio of FST positive and FST negative BPPV patients were similar, and we found FST to be invaluable alone in the diagnosis of BPPV.

When the FST results are interpreted, a turn greater than 45° to the either side, may imply a peripheral vestibular asymmetry. Although Unterberger stated that the rotation seen in the stepping test was always in same direction with the slow phase of nystagmus, several studies which examined the relation between the direction of turn and the side affected, reported no correlation.^10^, ^16^, ^17^, ^18^

The mechanism which triggers the vertigo spells in BPPV is well known, however the underlying pathology which results in the separation of otoconia from the neuroepithelium is obscure. Several factors such as aging, systemic diseases, microangiopathic changes and microtrauma are held responsible for the degeneration of the neuroepithelium.^19^ The cVEMP findings of unilateral BPPV patients were evaluated in the study of Karatas et al. and bilateral involvement of the macular neuroepithelium was detected.^20^ In our study we found no significant relationship between the direction of deviation in FST and the side affected by BPPV. The explanatory reason for this may be the asymmetrical bilateral degeneration of the macular neuroepithelium.

Although BPPV happens to be a benign disease with symptoms resolving quickly after CRMs, some cases may be resistant to standard treatment manuvers and the recurrences may be seen frequently. It is called intractable BPPV. Intractable BPPV is usually associated with inner ear diseases such as Meniere’s disease, sudden sensorineural hearing loss and inner ear trauma. 21, 22, 23 However some cases of intractable BPPV may be idiopathic and not secondary to inner ear diseases. Patients with history of Meniere’s disease and/or other labyrinthopathies, recent trauma and inner ear diseases were excluded from our study, only patients with idiopathic BPPV were recruited. In our study 45% of the cases had recurrent BPPV after 3 months of recovery and considered as intractable BPPV. There is no definite test or parameter to predict the prognosis of BPPV. A few studies evaluated the value of cervial/ocular VEMPs as a prognostic factor for BPPV but the results are ambiguous.^9^, ^24^, ^25^ The number of BPPV attacks and the treshold and amplitude of cVEMP in affected ears was found to be correlated, by Longo et al.^24^ The absence of cVEMP or oVEMP response was found to be more frequent in the recurrent BPPV cases, by Lee et al.^25^ Chang et al. stated that decreased cVEMP IAD ratio at the affected ear was associated with resistance to CRMs.^6^ However the recent meta-analysis of Oya et al. stated that the latencies of BPPV and control patients were indifferent and VEMPs were not suitable for the prediction of prognosis or severity of BPPV.^26^ Although we found FST to be invaluable alone in the diagnosis of BPPV, we considered that FST could be a valuable bedside test to predict the prognosis of BPPV. Our study showed that the need for multiple CRMs was significantly higher in the FST positive group. In addition, recurrence frequency was significantly higher in the FST positive group. Therefore, we think that as the severity of BPPV increases, the overall diagnostic performance of the FST improves.

BPPV is usually caused by the separation of otoconia and debris from the neuroepithelium of utricular or saccular macula. The freely floating otoconia in the semicircular canals provoke short-term nystagmus and vertigo (canalolithiasis). The features of nystagmus in canalolithiasis are a latency of 5–30 s, a burst of nystagmus lasting approximately 10 s, the direction of the nystagmus is about the axis of the affected canal, fatigueability due to the margination of the debris, a reversal of nystagmus on sitting. Rarely BPPV may be attributed to otoconia that are attached to the cupula of a semicircular canal, it is called cupulolithiasis.^12^ This load of otoconia makes the system sensitive to gravitational forces and the alterations in cupular deflection lead to pathological perception of motion. The typical features of nystagmus in cupulolithiasis are no latency, permanent nystagmus lasting more than one-minute, weak nystagmus (less than 5°/s), direction changing nystagmus. It is suggested that cupulolithiasis represents the more chronic form of BPPV. Furthermore, cupulolithiasis may be more common in patients who have gone untreated for a long time, giving the otoconia more time to stick to the cupula.^27^

The limitation of our study is that due to the rare incidence of cupulolithiasis we did not seperate the FST positive and FST negative groups into canalolithiasis and cupulolithiasis subgroups. Cupulolithiasis representing the more chronic form of BPPV, may have a role on increased vestibular asymmetry and therefore leading to positive results in FST. More studies should be performed to establish the relevance of FST results with BPPV cases having canalolithiasis or cupulolithiasis.

## Conclusion

The FST is a bedside test based on vestibulospinal reflex and widely used for the assessment of the labyrinthine function. Although the sensitivity of the FST in detecting mild/moderate unilateral vestibular dysfunction is poor, it may be valuable in the predicton of the prognosis of BPPV. As the severity of the disease increases, the overall diagnostic performance of the FST improves. Therefore, we suggest that the positive FST results in BPPV patients indicate poor prognosis, the need for multiple CRMs and the possibility of recurrences.

## Conflicts of interest

The authors declare no conflicts of interest.
